# RAGE contributes to persistent sepsis-induced muscle and mitochondrial alterations

**DOI:** 10.1038/s41598-025-28645-8

**Published:** 2025-11-28

**Authors:** Raphaël Romien, Alexandre Pierre, Sarah Ducastel, Arthur Dubech, Jérémy Lemaire, Gaëlle Grolaux, Marie Frimat, Benoit Brassart, Claire Bourel, Michael Howsam, Cécile Yelnik, Eric Boulanger, Raphaël Favory, Sebastien Preau, Steve Lancel

**Affiliations:** 1 Univ. Lille, Inserm, CHU Lille, Institut Pasteur de Lille, U1167 - RID-AGE - Facteurs de risque et déterminants moléculaires des maladies liées au vieillissement, 59000 Lille, France; 2 Division of Intensive Care, Hôpital Roger Salengro, CHU de Lille, 59000 Lille, France; 3 Division of Nephrology, Hôpital Claude Huriez, CHU de Lille, 59000 Lille, France; 4 Division of Geriatrics, Hôpital Albert Calmette, CHU de Lille, 59000 Lille, France

**Keywords:** Cell biology, Diseases, Immunology, Pathogenesis, Physiology

## Abstract

A majority of patients surviving sepsis develop muscle weakness. However, the underlying cellular and molecular pathways remain largely unexplored. To determine whether sepsis leads to long-term persistent muscular consequences and to identify the underlying mechanisms, we used a murine model of reanimated sepsis induced by intraperitoneal injection of a heterologous stool slurry. Muscles were analyzed 3 months later. The oxidative muscle exhibited reduced fatigue resistance and decreased mitochondrial respiration, without a corresponding reduction in mitochondrial OXPHOS proteins. Glycolytic and mixed muscle fibres were atrophied. Markers of oxidative and mitochondrial stress, as well as genes involved in mitochondrial fission, remained present 3 months after sepsis. Low-grade, but significant, muscular inflammation was also measured. Specifically, both the NLRP3 inflammasome and the receptor of Advanced Glycation End-products (RAGE) axis were upregulated. Interestingly, long-term sepsis-induced muscular consequences were not observed in RAGE knockout mice. Overall, we describe for the first time in mice that sepsis causes long-lasting muscle dysfunction after recovery, including mitochondrial alterations and low-grade inflammation, and that RAGE may represent a promising target to mitigate long-term muscle alterations induced by sepsis.

**Supplementary Information:**

The online version contains supplementary material available at 10.1038/s41598-025-28645-8.

## Introduction

Sepsis-induced organ dysfunction results from a dysregulated systemic immune response to host infection^1,2^. In 2017, sepsis affected almost 49 million people and caused around 11 million deaths^1,2^. Patients admitted to an intensive care unit (ICU) with septic shock have a mortality rate of around 40%^1^. ICU-acquired weakness (ICUAW) is a widespread complication affecting a majority of survivors^3–5^. The reduction in muscle force persists for several months after sepsis, seriously impacting the quality of life of survivors, affecting daily activities, leading to a deterioration in physical condition, and increasing the risk of long-term mortality^3,6^.

Skeletal muscle represents around 40% of human body mass and plays a key role in maintaining posture, locomotion, and the conversion of chemical energy into mechanical work. It is a major player in energy metabolism, participating in the storage of amino acids and carbohydrates, thermoregulation, and energy production during exercise through mitochondrial ATP production. Any skeletal muscle damage has immediate repercussions on independence, quality of life, and general health^7,8^.

It has been shown that human sepsis survivors have reduced muscle force and collagen deposition in skeletal muscle for up to 5 years after their admission to the ICU^9^. In addition, in mice, it has been shown that the muscle weakness observed in the first hours after sepsis onset can persist for up to one month^10–12^. Sepsis survivors show a decrease after sepsis in muscle cross-sectional area (CSA) and force that lasts up to 70 days in mice and 5 years in humans^13–15^. Mitochondrial dysfunction may represent the main cause of post-sepsis muscle weakness^14^. It was shown that 5 years after ICU admission for critical illness, survivors had muscle weakness associated with lower respiratory-chain complex activity, and abnormal expression of mitochondria-related RNA^15^.

Mitochondrial alterations include reduced respiration, increased production of reactive oxygen species (ROS) and reactive nitrogen species (RNS), as well as impaired mitochondrial quality control mechanisms^16,17^. Among these last, impairment of autophagy flux (involved in the removal and recycling of damaged cellular components) has recently been described^18^. Mitochondrial dynamics involve both fusion and fission to support energy production or isolate damaged mitochondria for elimination. Sepsis disrupts mitochondrial dynamics in muscles, leading to mitochondrial dysfunction and increased levels of proteins involved in mitochondrial fission^19,20^. Thus, during sepsis, the mitochondrial quality control pathways (autophagy and mitochondrial dynamics) are impaired, leading to the accumulation of damaged mitochondria. Dysfunctional mitochondria may release mitochondrial DNA (mtDNA), which is considered to be a damage-associated molecular pattern (DAMP). DAMPs are messengers inducing inflammatory responses relying on the NOD-like receptor family pyrin domain containing 3 (NLRP3) inflammasome^17^. This pathway is responsible for pro-caspase-1 activation, which allows pro-interleukin-1β (Il-1β) cleavage into active Il-1β^21^. Activation of caspase 1 (CSP-1) by the NLRP3 inflammasome during sepsis leads to pyroptosis and massive release of high-mobility group box protein (HMGB1), which is one of the lethal mechanisms of sepsis^22–24^. HMGB1 has a powerful inflammatory capacity, binding to RAGE to induce pyroptosis^25^, and has been shown to remain increased for at least 28 days post-sepsis in a Cecal Ligation and Puncture-induced (CLP) mouse model^23^.

However, these mechanisms associated with mitochondrial dysfunction and inflammation are mainly described during the acute phase of sepsis, and knowledge of the long-term impacts of sepsis on skeletal muscle remains limited. Our study aimed to use a long-term sepsis survivor mouse model to study skeletal muscle-related alterations by evaluating muscle function and phenotype, mitochondrial function, inflammation, and the role of RAGE in modulating long-term sepsis-induced muscle alterations.

## Results

### Sepsis does not change body composition in the long term

**Fig. 1:**
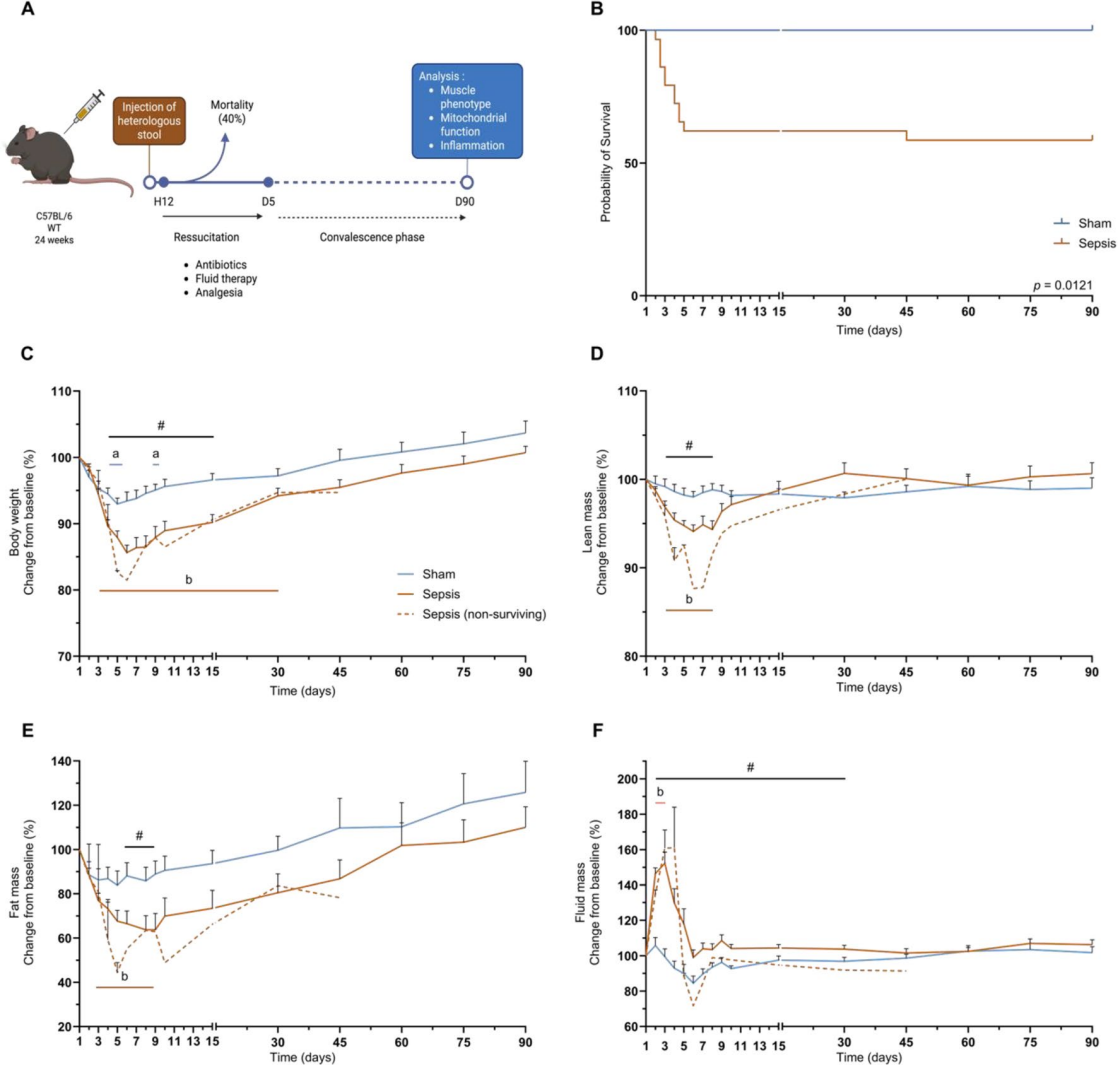
Sepsis does not change body composition in the long term. (**A**) Diagram of the protocol enabling long-term analysis of mice surviving sepsis. The mice are injected with heterologous stool, and a resuscitation phase delayed by 12 h is performed for 5 days. During the resuscitation phase, the mice are injected with antibiotics, analgesia, and hydration. (**B**) Kaplan-Meier curves for Sham (*n* = 12) and Sepsis mice (*n* = 29). One mouse was excluded based on the exclusion criteria. Changes compared with baseline in weight (**C**), lean mass (**D**), fat mass (**E**) and fluid mass (**F**) were measured over 90 days for Sham (*n* = 12), Sepsis (*n* = 17) and sepsis non-surviving (*n* = 12). Statistical comparisons are between Sham vs. Sepsis (#), Sham vs. baseline (a), Sepsis vs. baseline (b) (**B**, **C**, **D**, **E**), and Sham vs. Sepsis (*). Data were analysed with log-rank test (**B**), mixed-effects model (REML) (**C**, **D**, **E**, **F**). * *p* < 0.05.

Sepsis was induced in mice by injection of a heterologous stool slurry, followed by a delayed resuscitation phase starting 12 h later and lasting for 5 days. Resuscitation was performed by administration of antibiotics, hydration, and analgesia. Mice were analysed 3 months after sepsis induction (Fig. 1A). During the acute phase of sepsis, corresponding to the first 10 days, mortality was around 38% in the Sepsis group. One mouse was excluded based on the exclusion criteria (Fig. 1B). One death occurred in the Sepsis group during the convalescence period (10 to 90 days; Fig. 1B). Sepsis mice lost up to 8% (*p* = 0.0004) more weight than Sham mice between days 4 and 15. From day 3 after sepsis induction, Sepsis mice lost up to 14.4% of their initial body weight (*p* < 0.0001), and the loss persisted until day 30 (Fig. 1C). For the lean mass, we found a decrease of up to 4.5% (*p* = 0.0009) in the Sepsis group compared with the Sham group between days 3 and 8 (Fig. 1D). Compared to baseline, lean mass in the Sepsis group decreased by up to 5.9% (*p* < 0.0001 Fig. 1D). Between day 6 and 9, the Sepsis group had 25.1% (*p* = 0.0131) less fat mass than the Sham group, a reduction of up to 36.3% (*p* = 0.0040) compared with pre-induction baseline in the Sepsis group (Fig. 1E). Fluid mass increased by up to 52.8% (*p* < 0.0001) in Sepsis compared with Sham mice from day 2 to 30, but was higher than baseline only between days 2 and 3, increasing to 52.3% (*p* < 0.0001), likely reflecting the resuscitation phase during which saline solution was administered subcutaneously, and possibly also reduced fluid losses due to transient renal dysfunction and/or increased capillary permeability (Fig. 1F). In contrast, no significant changes in any of these parameters remained between Sham and Sepsis survivors 3 months later (Fig. 1).

### Sepsis leads to long-term oxidative muscle fatigability and atrophy of skeletal muscle fibres

**Fig. 2:**
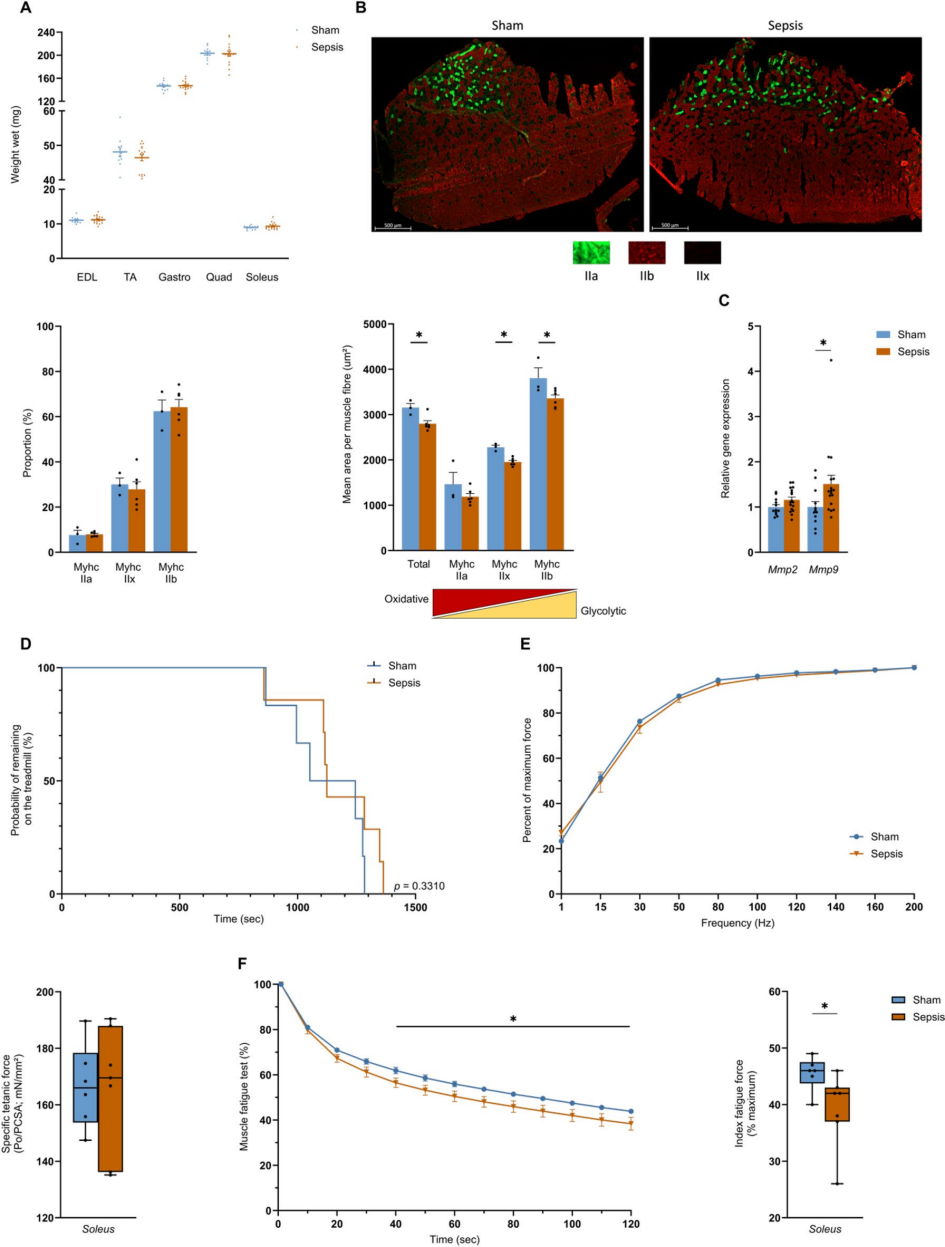
Sepsis induces long-term muscle alterations in surviving mice. (**A**) wet weights of *EDL*, *TA*, *gastrocnemius*, *quadriceps*, and *soleus* for Sham (*n* = 12) and Sepsis (*n* = 17) mice. (**B**) representative fluorescence microscopy images of *TA* from Sham (left) and Sepsis (right) mice immunostained for type IIa (green), IIb (red), and IIx fibres (unlabelled). The total surface area of all and each fibre type per muscle section was measured and quantified by the MuscleJ2 (Fiji) plugin for the Sham (*n* = 3) and Sepsis (*n* = 6) groups. (**C**) gene expression of Mmp2 and Mmp9, normalised to the Sham group (Sham mice *n* = 12, Sepsis mice *n* = 17). (**D**) Kaplan-Meier curves of the probability of a Sham (*n* = 6) or Sepsis (*n* = 7) mouse running. (**E**) percentage of maximum contractile force according to frequency. Whisker box plots representing the median, lower and upper quartiles, and minimum and maximum values. The data represent *soleus*-specific tetanic force production in the Sham (*n* = 6) and Sepsis (*n* = 7) groups. (**F**) evolution of *soleus* fatigue (expressed as a percentage relative to the initial force) over time during 2 min. Whisker box plots representing the median, lower and upper quartiles, and minimum and maximum values. The data represent the percentage of *soleus* fatigue reached after 2 min of contraction compared to the initial value in the Sham (*n* = 6) and Sepsis (*n* = 7) groups. Data are expressed as mean with SEM. Statistical comparisons are between Sham vs. Sepsis (*). Data were analysed with log-rank test (**D**), with Mann-Whitney test (**A**, **B**, **C**, **E**, **F**) or mixed-effects model (REML) (**E**, **F**), t-test (**A**, **C**). * *p* < 0.05.

In line with the absence of changes in the body composition, muscle wet weight of the *extensor digitorum longus* (*EDL*), the *tibialis anterior* (*TA*), the *gastrocnemius*, the *quadriceps*, and the *soleus* were not different between the Sham and the Sepsis groups at 3 months (Fig. 2A). Analysis of the distribution of fibre type in the *TA* showed no changes in either group (Fig. 2B). However, the total CSA was significantly reduced from 3153 to 2798 µm^2^ (*p* = 0.0476) 3 months after sepsis (Fig. 2B). In particular, fibre types IIb and IIx decreased in the Sepsis group from 3804 to 3355 µm^2^ (*p* = 0.0476) and from 2279 to 1953 µm^2^ (*p* = 0.0238), respectively, by the end of the experiment (Fig. 2B). Along with these changes, the Sepsis group showed a significant 50% (*p* = 0.0267) increase in matrix metalloproteinases 9 (*Mmp9*) expression (Fig. 2C). No changes were found in the expression of the main genes involved in cell cycle arrest (Fig. 1SA). When we assessed muscle function using a treadmill test and an ex vivo test. Mice in both the Sham and the Sepsis groups ran for similar duration on the treadmill, with average running times of 1119.5 s and 1171.7 s, respectively (Fig. 2D). Both groups showed similar specific tetanic force, and there were also no differences in percentage force between the two groups according to the frequency applied, from 1 to 200 Hz (Fig. 2E). The *soleus* fatigability test showed that the Sepsis group was more fatigable from 40 s to the end of the test, with a mean difference of 5.5% (*p* = 0.0395) of fatigability, and, by the end of the fatigue test, the Sepsis group exhibited 14% (*p* = 0.0315) higher muscle fatigue than the Sham group (Fig. 2F).

### Sepsis is responsible for long-term, persistent mitochondrial alterations in skeletal muscles

**Fig. 3:**
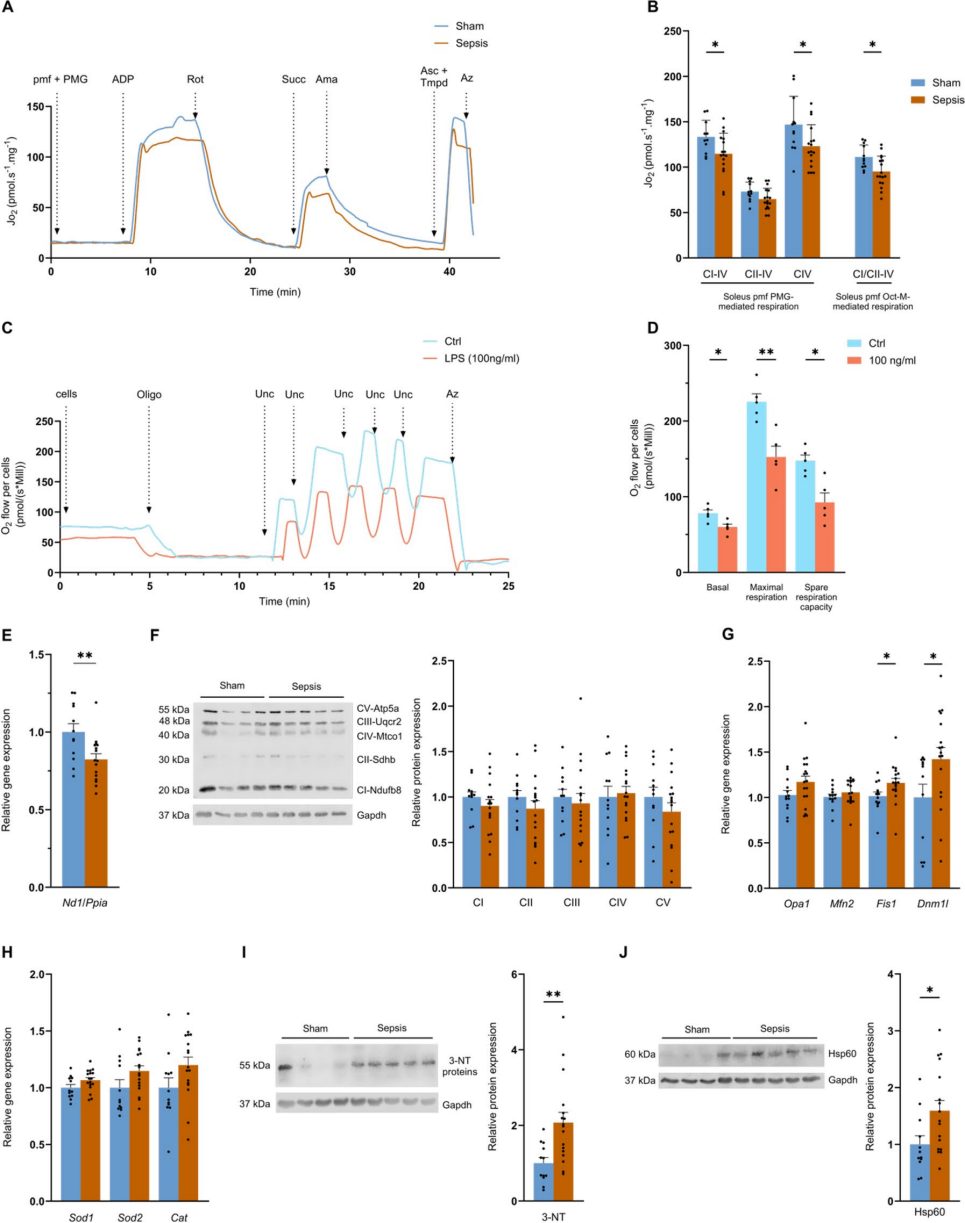
Sepsis induces persistent long-term mitochondrial dysfunction. (**A**) Representative curve of the pyruvate-glutamate-malate (PMG) protocol assessing oxygen consumption (Jo_2_) of permeabilised *soleus* muscle fibres (pmf) from Sham (blue) and Sepsis (orange) mice by high-resolution respirometry using successive injections: pyruvate (5 mM), malate (2 mM), glutamate (10 mM) (PMG; ADP (5 mM) (CI-IV oxidative phosphorylation state (OXPHOS) driven by complex I); rotenone (Rot, 0.5 µM) and succinate (Succ, 10 mM) (CII-IV OXPHOS state driven by complex II); antimycin A (Ama, 2.5 µM), ascorbate (Asc, 2 mM) and TMPD (0.5 mM) (CIV OXPHOS state driven by CIV) and sodium azide (Azd, 100 mM). (**B**) pmf Jo_2_ data from *soleus* of Sham (*n* = 12) and Sepsis (*n* = 17) mice in PMG protocols for CI-driven OXPHOS states of respiration at CIV, CII-driven at CIV and CIV and by octanoyl-L-carnitine-malate (Oct-M) protocol for CI- and CII-driven OXPHOS state of respiration at CIV. (**C**) Representative curve of the oxygen consumption protocol performed on ctrl (blue) or LPS-treated (pink) myotubes by high-resolution respirometry following successive injections of oligomycin (oligo, 10 µM), uncoupler (Unc, 1 µM), and sodium azide (azd, 100 mM). (**D**) oxygen consumption of muscle cells treated with placebo (ctrl) or LPS at 100 ng/ml (LPS) from basal respiration with no substrate or inhibitor supplementation, uncoupled maximal respiration, and free reserve respiration (maximal respiration minus basal respiration) (*n* = 5 per group). (**E**) amount of mtDNA measured by normalisation of *Nd1* mRNA on *Ppia* mRNA in *quadriceps* in Sham (*n* = 12) and Sepsis (*n* = 16). (**F**) protein expression analysis of Ndufb8 (CI), Sdhb (CII), Uqcr2 (CIII), Mtco1 (CIV), Atp5a (CV), and Gapdh in *quadriceps* for Sham (*n* = 12) and Sepsis (*n* = 17). mRNA level measured by Quantigene of *Sod1*, *Sod2* and *Catalase* (*Cat*) in *quadriceps* for Sham (*n* = 12) and Sepsis (*n* = 17) groups. (**G**) mRNA level measured by Quantigene of *OPA1*, *Mfn2*, *Fis1* and *Dnm1l* in *quadriceps* for Sham (*n* = 12) and Sepsis (*n* = 17) groups. (**H**) mRNA level measured by Quantigene of *Sod1*, *Sod2*, and *Cat* for Sham (*n* = 12) and Sepsis (*n* = 17) groups. (**I**) protein expression analysis of 3 nitrotyrosine (3-NT) and Gapdh in *quadriceps* for Sham (*n* = 12) and Sepsis (*n* = 17). (**J**) protein expression analysis of Hsp60 and Gapdh in *quadriceps* for Sham (*n* = 12) and Sepsis (*n* = 17). Data are expressed as mean with SEM. Statistical comparisons between Sham vs. Sepsis (*). Data were analysed with a t-test (**B**, **D**, **E**, **F**, **G**, **I**, **J**) or a Mann-Whitney test (**G**, **F**). * *p* < 0.05, ** *p* < 0.01.

Permeabilised fibres from the *soleus* of surviving Septic mice had a ~ 14% (*p* = 0.0312) lower oxygen consumption (JO_2_) for oxidative phosphorylation (OXPHOS) respiration driven by complex I to IV (Fig. 3A). Complex IV JO_2_ also decreased by around 16% (*p* = 0.0274) in the Sepsis group, from 146.8 at baseline to 123.1 pmol/s/mg at 3 months (Fig. 3B). Finally, the Oct-M protocol used for assessing fatty acid-mediated respiration also showed a decrease of around 14% (*p* = 0.0109), with a mean of 111.2 pmol/s/mg for the Sham group and 95.2 pmol/s/mg for the Sepsis group (Fig. 3B).

To examine whether prolonged infection can trigger mitochondrial dysfunction specifically in muscle cells themselves, we conducted a supplementary experiment where we treated C2C12 muscle cells with chronic (14 days) and low-dose (100 ng/mL) lipopolysaccharide (LPS). In the LPS-treated myotubes, basal respiration decreased by 23.2% (*p* = 0.0166), from 78 to 59.9 pmol/(s*Mill), and maximal respiration by 32.4% (*p* = 0.0035) from 225.5 to 152.5 pmol/(s*Mill), while the spare respiratory capacity was decreased by 37.2% (*p* = 0.0056), from 147.4 to 92.6 pmol/(s*Mill) (Fig. 3D).

Returning to the mice, the amount of mtDNA was reduced by almost 18% (*p* = 0.0092) in the Sepsis group compared with the Sham group (Fig. 3E). However, we did not detect any significant changes in the respiratory chain protein expression (Ndufb8, Sdhb, Uqcr2, Mtco1, and Atp5a) (Figs. 3F, S2).

We also explored mitochondrial quality control pathways. While levels of mitochondrial fusion-related genes *Opa1* and *Mfn2* were not different between groups, levels of mitochondrial fission-related genes, *Fis1* and *Dnm1l*, were increased in Sepsis by 16% (*p* = 0.0197) and 42.1% (*p* = 0.0416), respectively (Fig. 3G). No differences, except a moderate increase in the *Park2* and *Lamp2* genes in the Sepsis group (*p* < 0,05), were found in autophagy/mitophagy gene expression (Fig. S1). Regarding oxidative stress, despite a 2-fold (*p* = 0.0016) increase in the 3-nitrotyrosine in Sepsis compared with Sham mice (Figs. 3I, S3), the mRNA levels encoding the antioxidant enzymes *Sod1*, *Sod2*, and *Cat* were not different (Fig. 3H). Finally, Hsp60 in Sepsis mice (a mitochondrial chaperone) increased by 59.2% (*p* = 0.0251) compared with Sham mice (Figs. 3J, S4).

### The NLRP3 and RAGE pathways remain activated 3 months after sepsis induction

**Fig. 4:**
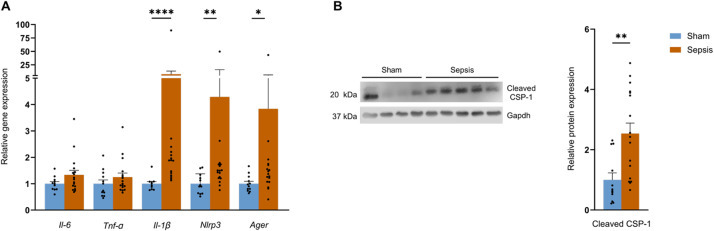
NLRP3 and RAGE-dependent inflammation persists in mouse survivors 3 months after sepsis. (**A**) mRNA levels of *Il-6*, *TNF-α*, *Il-1β*, *Nlrp3*, and *Ager* by Quantigene in *quadriceps* for Sham (*n* = 12) and Sepsis (*n* = 17). (**B**) protein expression analysis of cleaved caspase 1 (cleaved CSP-1) and Gapdh in *quadriceps* in Sham (*n* = 12) and Sepsis (*n* = 17). Data are expressed as mean with SEM. Statistical comparisons between Sham vs. Sepsis (*). Data were analysed with a t-test (**A**, **B**) or Mann-Whitney test (**A**). * *p* < 0.05, ** *p* < 0.01, *** *p* < 0.001.

Since inflammation contributes to the pathogenesis of sepsis^20,26^ and may participate in mitochondrial dysfunction, we assessed skeletal muscle gene expression of major pro-inflammatory pathways. While no differences were found for *Il-6* and *TNF-α* between both groups, *Nlrp3* mRNA increased 4-fold (*p* = 0.0043) and *Il-1β* mRNA increased 7.5-fold in Sepsis versus Sham mice (*p* < 0,0001). In line with this finding, cleaved caspase CSP-1, an effector protein of the activation and assembly of the NLRP3 inflammasome, was 2.5-fold higher in the Sepsis group compared with the Sham group (*p* = 0.0025; Figs. 4B, S5). Finally, *Ager* gene expression remained 3.8-fold higher (*p* = 0.0141) in the Sepsis group at 3 months (Fig. 4A).

### *Ager*^*−/−*^ mice do not develop mitochondrial dysfunction and long-term inflammation after sepsis

**Fig. 5:**
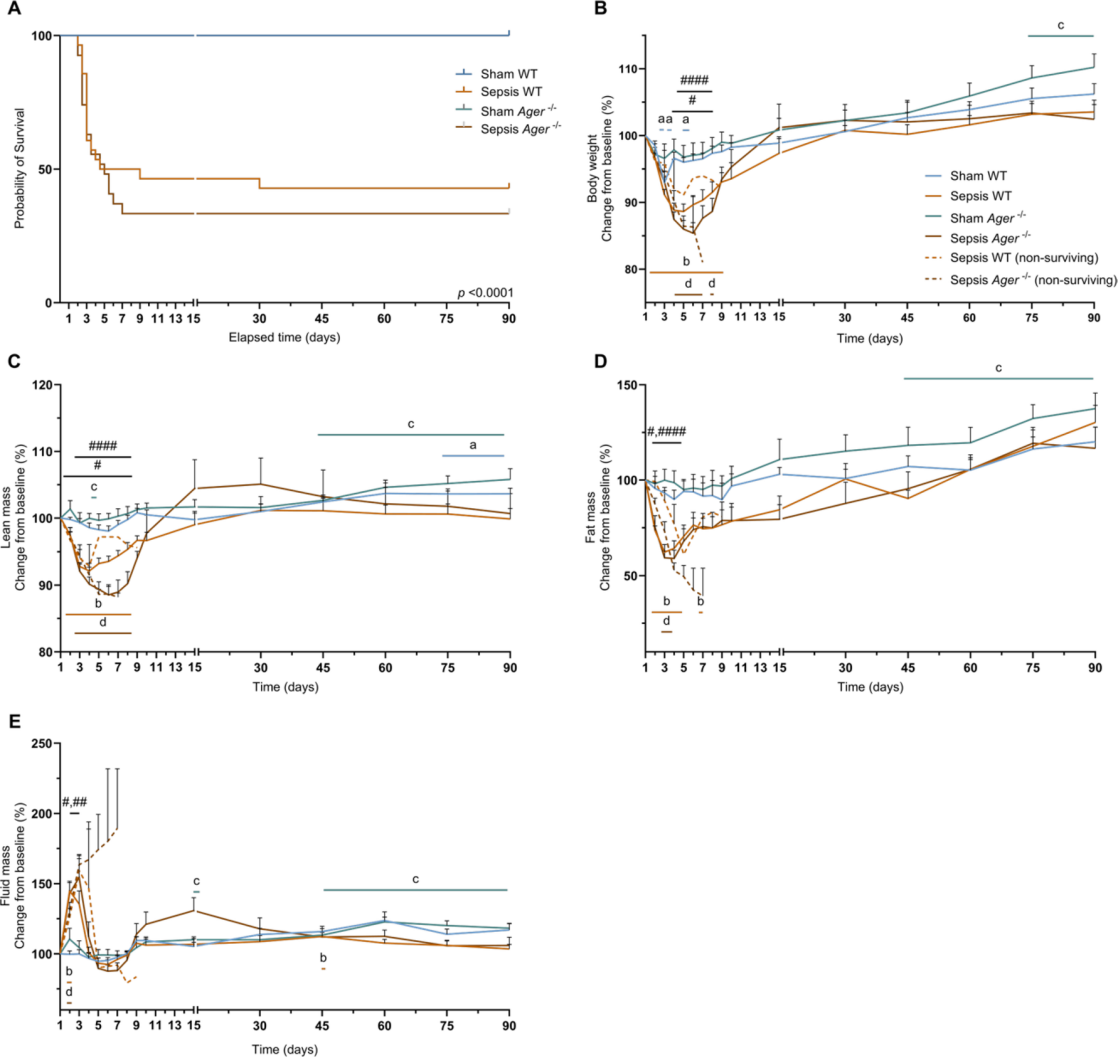
Survival and body composition of Ager^-/-^ mice do not differentiate from WT mice. (**A**) Kaplan-Meier curves for Sham WT (*n* = 12), Sepsis WT (*n* = 28), Sham *Ager*^**-/-**^ (*n* = 12), and Sepsis *Ager*^**-/-**^ (*n* = 27) groups. (**B**, **C**, **D**, **E**) baseline changes in weight, fat mass, lean mass and fluid mass were measured over 90 days for Sham WT (*n* = 12), Sepsis WT (*n* = 12), Sham *Ager*^**-/-**^ (*n* = 12), Sepsis *Ager*^**-/-**^ (*n* = 7), Sepsis WT non-surviving (*n* = 15) and Sepsis *Ager*^**-/-**^ non-surviving (*n* = 18). Statistical comparisons are between Sham WT vs. Sepsis WT (#), Sham *Ager*^**-/-**^ vs. Sepsis *Ager*^**-/-**^ (####), Sham WT vs. baseline (a), Sepsis WT vs. baseline (b), Sham *Ager*^**-/-**^ vs. baseline (c) and Sepsis *Ager*^**-/-**^ vs. baseline (d) (**B**, **C**, **D**, **E**). Data were analysed with a log-rank test (**A**) and mixed-effects model (REML) (**B**, **C**, **D**, **E**). * *p* < 0.05.

To evaluate a potential link between inflammation and mitochondrial dysfunction observed three months after sepsis, we also examined *Ager*^−/−^ mice. Mortality was similar between the Sepsis WT and *Ager*^−/−^ groups, around 60% (Fig. 5A). In the long term, increases in lean body mass were observed in Sham WT (Fig. 5C), and in all body composition parameters in Sham *Ager*^⁻/⁻^ mice (Fig. 5B–E), whereas no differences in body weight, lean mass, fat mass, or fluid mass were found between Sepsis WT and Sepsis *Ager*^⁻/⁻^ mice at any time point (Fig. 5).

**Fig. 6:**
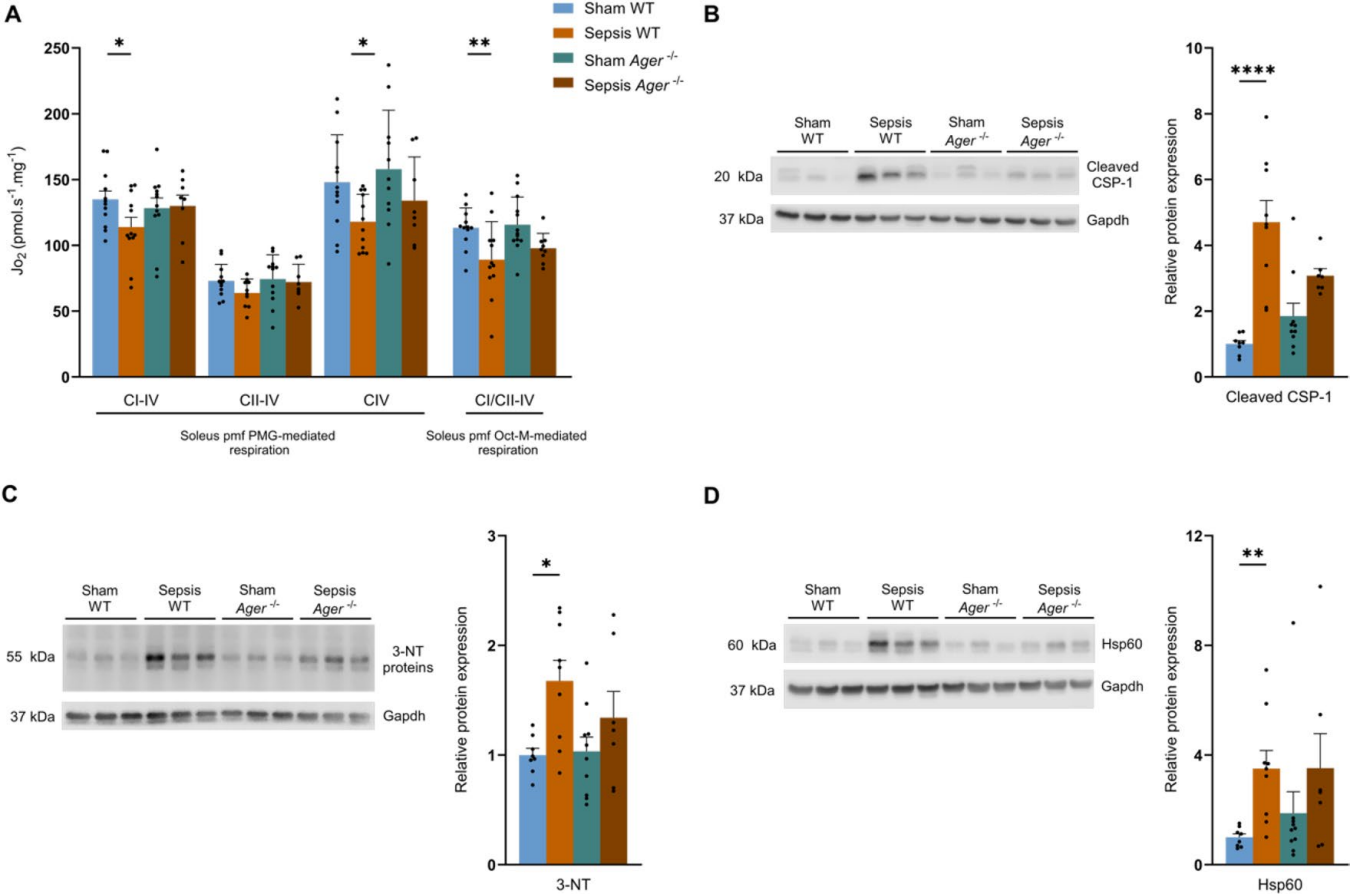
*Ager*^*-/-*^ sepsis mice survivors do not develop mitochondrial dysfunction and inflammation. (**A**) *soleus* pmf Jo_2_ data from Sham WT (*n* = 12), Sepsis WT (*n* = 12), Sham *Ager*^**-/-**^ (*n* = 12), and Sepsis *Ager*^**-/-**^ (*n* = 9) mice in PMG protocols for OXPHOS states of respiration driven by CI to CIV, CII to CIV, and CIV, and by the octanoyl-L-carnitine-malate (Oct-M) protocol for the OXPHOS state of respiration conducted by CI and CII to CIV. (**B**) protein expression of cleaved caspase-1 (cleaved CSP-1) and Gapdh in *quadriceps* in Sham WT (*n* = 8), Sepsis WT (*n* = 9), Sham *Ager*^**-/-**^ (*n* = 9), and Sepsis *Ager*^**-/-**^ (*n* = 7). (**C**) protein expression of 3-nitrotyrosine (3-NT) and Gapdh in *quadriceps* in Sham WT (*n* = 8), Sepsis WT (*n* = 9), Sham *Ager*^**-/-**^ (*n* = 9), and Sepsis *Ager*^**-/-**^ (*n* = 7). (**D**) protein expression of Hsp60 and Gapdh in *quadriceps* in Sham WT (*n* = 8), Sepsis WT (*n* = 9), Sham *Ager*^**-/-**^ (*n* = 9), and Sepsis *Ager*^**-/-**^ (*n* = 7). Data are expressed as mean with SEM. Statistical comparisons between Sham WT vs. Sepsis WT vs. Sham *Ager*^**-/-**^ vs. Sepsis *Ager*^**-/-**^ (*). Data were analysed with a one-way ANOVA test with Fisher’s LSD post-hoc (**A**), Sidak post-hoc (**C**), or a Kruskal-Wallis test with Dunn’s post-hoc test (**B**, **D**). * *p* < 0.05, ** *p* < 0.01, ****p < 0.0001.

Mitochondrial respiration was lower in the Sepsis WT group compared with the Sham WT group, decreasing from 134.9 J_O2_ to 113.9 J_O2_ (*p* = 0.0424) for complex I-mediated respiration and 147.9 J_O2_ to 117,8 (*p* = 0.04) J_O2_ for complex IV-mediated respiration in the PMG protocol. Similarly, fatty acid-mediated respiration showed a decrease from 113.2 J_O2_ to 89.1 J_O2_ (*p* = 0.0066) in Sepsis WT (Fig. 6A). Sepsis *Ager*^−/−^ and Sham *Ager*^−/−^ mice showed no differences, whatever the state of respiration and protocol used (Fig. 6A). Expression of cleaved CSP-1 increased by ~ 5-fold (*p* < 0.0001) in the WT Sepsis group compared with the WT Sham group. In the *Ager*^−/−^ Sepsis group, cleavage CSP-1 was not different from the *Ager*^−/−^ Sham group (Figs. 6B, S6). As in Fig. 3I-NT protein levels were increased by ~ 70% (*p* = 0.0241) in Sepsis WT mice compared with Sham WT mice, but there was no difference between Sepsis *Ager*^−/−^ and Sham *Ager*^−/−^ mice, nor between Sepsis WT and Sepsis *Ager*^−/−^ animals (Figs. 6C, S7). Finally, while the expression of Hsp60 was higher by 3.5-fold (*p* = 0.0077) in the Sepsis WT group compared with the Sham WT group, no differences between Sepsis *Ager*^−/−^ and Sham *Ager*^−/−^ groups, nor Sepsis WT and Sepsis *Ager*^−/−^, were observed for this parameter (Figs. 6D, S8).

## Discussion

We used a murine sepsis survivor model based on previously validated experimental approaches by Owen et al. and Pierre et al., and we characterised this model in the long-term, i.e. 3 months^11,12,27^. Mice develop muscle weakness, mitochondrial dysfunction, and inflammation, alterations that are also observed following sepsis in the early phase^10,11,26,28^. Although few long-term studies are available, these alterations have also been observed in the mid-to-long term following sepsis in humans^9,15,29^ and mice^11,14,27,30^. These findings confirm our mouse model as a reliable and translationally relevant tool for the study of chronic muscle alterations following sepsis. Interpretations regarding muscle-specific effects should be made with caution, as our conclusions are based on alterations observed across different skeletal muscles with distinct metabolic profiles. Nevertheless, these convergent findings indicate a generalised impact of sepsis on multiple muscle types.

Our investigation in sepsis survivor mice shows similar persistent alterations in muscle phenotype. First, we observed a decrease in the CSA of IIx and IIb fibres of *TA* muscle. This result should be interpreted cautiously given the small number of sham samples. Although overall muscle wet weight remained unchanged, this likely results from edema, inflammation, or fibrosis, as well-documented in sepsis, all of which can mask actual muscle atrophy^9,12,15^. Nevertheless, studies have shown that sepsis survivors have a reduction in CSA, which is correlated with a reduction in muscle force^31,32^. In the murine sepsis model, treadmill performance and maximal tetanic force were not significantly altered despite muscular atrophy. This observation aligns with findings reported by Dos Santos et al., Owen et al., and Kingren et al., who demonstrated an absence of correlation between muscle mass or atrophy and strength in sepsis survivors. Such structure-function decoupling underscores the importance of muscle quality rather than muscle mass alone in the recovery process following sepsis^11,14,33^. Although oxidative type IIa fibres did not exhibit signs of atrophy, they displayed reduced fatigue resistance, suggesting a qualitative alteration of muscle function. Since oxidative muscles rely predominantly on mitochondrial oxidative phosphorylation (OXPHOS) for energy production, we next investigated mitochondrial respiration in the soleus^34^. Using permeabilised *soleus* fibres, we found that mitochondrial respiration based on the two main metabolic pathways (glycolysis and fatty acid oxidation) was impaired, especially in the respiration mediated by complexes I and IV. Our complementary in vitro experiments on C2C12 muscle cells further support our in vivo findings, indicating mitochondrial dysfunction within muscle cells. Several studies have reported impaired mitochondrial respiration in skeletal muscle and the diaphragm following sepsis^11,19,27^. In our study, a deeper investigation of mitochondrial defects revealed a reduction in mtDNA content, increased transcription of mitochondrial fission-related genes, and evidence of both mitochondrial and oxidative stress. Although the reduction in mtDNA content was approximately 18%, this did not significantly affect the protein levels of the respiratory complexes. In the murine sepsis model, the reduction in mtDNA content is moderate, and protein levels remain unchanged. Several studies have shown that only severe mtDNA depletion (above 50%) affects mitochondrial mRNA expression, protein abundance, and respiratory function^35,36^. Dos Santos et al. demonstrated that 6 months after ICU discharge, survivors showed no changes in mitochondrial content or population size^33^. These observations support the hypothesis that the mitochondrial dysfunction observed in the murine sepsis model may result primarily from reduced mitochondrial efficiency rather than a decrease in mitochondrial number. However, this interpretation should be viewed with caution, as human data and preclinical studies may differ regarding the regulation of mitochondrial dynamics. Previous studies have also reported that excessive mitochondrial fission is linked to impaired mitochondrial efficiency in skeletal muscle after sepsis in mice^20^. We found an increase in pro-fission-related genes Drp1 and Fis1, which could promote aberrant mitochondrial fission and may contribute to the impaired long-term efficiency of mitochondrial respiration after sepsis. Further experiments are required to fully characterise this mitochondrial quality control pathway. Finally, the increased levels of mitochondrial and oxidative stress markers, such as Hsp60 and 3-NT, suggest that mitochondria are stressed and in a pro-oxidant environment. This indicates that the early nitro/oxidative stress observed after 10 days of sepsis endures long afterwards^11,12^. Moreover, the accumulation of 3-NT, known to reflect oxidative damage to mitochondrial complex I, was accompanied by a decrease in complex I-mediated respiration^11,27,37,38^. These findings further support the hypothesis of altered mitochondrial quality in sepsis survivors. Finally, these data are consistent with previous work in our laboratory and with Owen et al., who observed an increase in 3-NT labelling two weeks after sepsis in the muscle^11^. It is noteworthy that Hsp60, through its chaperone function within the mitochondrial unfolded protein response (UPRmt), contributes to the stability of respiratory complex proteins. Under mitochondrial dysfunction and oxidative stress conditions, it assists in the proper folding of SOD2, a key mitochondrial enzyme involved in antioxidant defense. Thus, Hsp60 plays an important role in managing oxidative stress and limiting the resulting damage^39,40^. When released extracellularly, it also acts as a DAMP that stimulates NLRP3-mediated inflammation^41–43^. These data therefore suggest that the increase in Hsp60 may reflect both an adaptive response to mitochondrial stress triggered by oxidative damage and respiratory dysfunction, as indicated by 3-NT positive proteins and its contribution to inflammation via NLRP3 inflammasome activation.

We subsequently sought to identify key pro-inflammatory mediators involved in sepsis, including TNF-α, IL-6, and the NLRP3 and RAGE signalling pathways^20,24,44^. Our results showed transcriptional activation of the NLRP3 pathway and the resultant IL-1β, as well as the cleaved effector protein CSP-1. It has been shown that the NLRP3 pathway is involved in sepsis and that its inhibition leads to improvement of muscle atrophy and inflammation^26^. It has also been shown that NLRP3 can be activated by DAMPs from mitochondria, through mtDNA leakage or ROS production^45,46^. We also observed that the *Ager* transcript, encoding for the RAGE receptor, was 4-fold higher in muscle from sepsis survivors, while elevated levels of key RAGE ligands were found during sepsis and several months afterward^20,47–49^. RAGE is involved in various deleterious processes – features of aging – including disruption of mitochondrial function, chronic inflammation (inflammaging), and senescence^50–52^. In our experimental model, genes associated with senescence did not show significant change; however, we observed upregulation of Mmp9, a gene involved in inflammation and fibrosis^53^. Thus, sepsis leads to long-term alterations that are also found in aging muscle and in sarcopenia^54,55^. Based on these observations, sepsis may contribute to accelerated aging of skeletal muscle in the long term, though further work is needed to better characterise and understand the mechanisms involved in post-septic muscle aging. Circulating metabolic and inflammatory parameters were not assessed in this study, and a systemic contribution to the observed muscular alterations cannot be completely excluded, as several studies have reported persistent metabolic and inflammatory disturbances following sepsis^29,56,57^. However, Owen et al. observed normalisation of circulating inflammatory markers despite persistent skeletal-muscle and mitochondrial alterations similar to those described here, suggesting a muscle-specific component to these long-term effects^11^.

To determine whether RAGE may represent a central point linking mitochondrial dysfunction and inflammation, we tested whether *Ager*^−/−^ mice were protected from the two main long-term consequences of sepsis, i.e. mitochondrial dysfunction and inflammation. Mortality did not differ between *Ager*^−/−^ and WT mice during sepsis. Several studies have reported either protective or deleterious effects of RAGE inhibition on survival following infection, highlighting the heterogeneous impact of RAGE deletion. In the murine sepsis model, the standardised antibiotic therapy likely compensated for the loss of RAGE-dependent inflammatory responses, while *Ager*^−/−^ mice may have developed additional compensatory immune mechanisms preserving survival. Three months after induction of sepsis, and in contrast to WT mice, *Ager*^−/−^ mice had not developed mitochondrial dysfunction or inflammation. This is consistent with reports indicating that RAGE inhibition may improve mitochondrial and muscle function during sepsis in mice^20,50^. Those beneficial effects of RAGE invalidation on aging-related parameters are consistent with previous studies^50,51^ and show that activation of the NLRP3 pathway primarily depends on NF-κB signaling, which is itself stimulated by RAGE^58,59^. Activation of NLRP3 has been linked to both reduced muscle function and muscle atrophy^60–62^. Thus, the absence of Csp-1-mediated inflammation in *Ager*^*−/−*^ mice may indicate a potential improvement in muscle function after sepsis. However, we did not quantify Il-1β, the final effector cytokine that directly affects muscle tissue. Future studies will need to confirm Il-1β expression and clarify the signaling pathways underlying its activation, as well as its reduction in the *Ager*^*−/−*^ model to validate this hypothesis.

In parallel, no studies had reported such a long-term protective effect on sepsis-induced persistent mitochondrial dysfunction and NLRP3-CSP1-mediated inflammation. Future studies should include direct assessments of muscle contractile performance in *Ager*^*−/−*^ mice to confirm whether the observed molecular and mitochondrial protection translates into preserved muscle function. Finally, further investigations are required to establish the underlying molecular mechanisms and to determine whether pharmacological RAGE inhibition may prevent muscular damage induced by sepsis.

## Conclusion

We first established a long-term survivor mouse model of sepsis that faithfully reproduces the various alterations observed in skeletal muscle several months after the initial infection. Long-term sepsis survivors exhibit muscle weakness associated with mitochondrial dysfunction and inflammation. RAGE invalidation prevents sepsis-induced mitochondrial dysfunction and inflammation in mice surviving sepsis. Development of specific RAGE inhibitors may represent an interesting strategy to prevent long-term muscular consequences triggered by an acute inflammation.

## Materials and methods

### Murine model of sepsis

Wild-type (WT) C57BL/6J mice (Charles Rivers Laboratories, Saint Germain Nuelles, France) and WT and *Ager*^−/−^ littermates obtained from *Ager*^+/−^ (from Pr. A.M. Schmidt, New York University) mice crossing in our animal facility were housed in specific pathogen-free (SPF) environment, with 12 h/12 h light and dark cycles and a temperature at 21 °C. Mice had free access to food and water. Sepsis was induced by intraperitoneal (IP) injection of 360 µL of a heterologous stool suspension prepared in PBS-glycerol 10%. Sham mice underwent the same resuscitation procedures, including antibiotics, analgesia and subcutaneous hydration, but received an intraperitoneal injection of PBS-glycerol 10% instead of stool. The resuscitation phase was delayed by 12 h and was performed every 12 h for 5 days. Mice received IP analgesia (buprenorphine, 0.1 mg/kg), antibiotics (imipenem-cilastatin, 40 mg/kg), and subcutaneous hydration (NaCl 0.9%, 25 mL/kg). Temperature (Lasergrip 774, Etekcity, Anaheim, USA), weight, and clinical condition (murine sepsis score MSS^12^ were monitored every 12 h until day 10. Sepsis mice were excluded if, 12 h after induction, their MSS was < 10/28 or their body temperature remained > 30 °C, indicating insufficient development of the septic state. These animals were excluded from the sepsis cohort and thus neither considered as survivors or non-survivors. Sepsis mice were excluded if, 12 h after induction, their MSS was less than 10/24 or the temperature was greater than 30 °C. Mice were immediately euthanised by cervical dislocation if limit points (MSS > 24/28, a total absence of reaction following stimulation by the experimenter, and a weight loss greater than 20% compared to the initial body weight) were reached. These animals were counted as non-survivor. After the resuscitation phase, the mice were housed in the animal facility and euthanised by cervical dislocation 3 months after the induction of sepsis (average weight 35 g). For each mouse, the wet weight of each muscle was measured using a high-precision balance (SM 425i, VWR, Radnor, PA, USA). All animal procedures and methods were approved by the Lille ethics committee (Animal Experimentation Ethics Committee CEEA75, Authorization number APAFIS #43559-2023050417009712), in accordance with our national and institutional regulations, and with ARRIVE guidelines^63^.

### Longitudinal monitoring of body composition

Body composition was analysed by time-domain nuclear magnetic resonance (TD-NMR) in animals every 24 h for 5 days from the induction of sepsis (Minispec fl50, 7.5 Mhz, Bruker, Billerica, MA, USA). Analysis was repeated every 15 days until sacrifice. Data collected included lean mass, fat mass, and fluid mass.

### Cross-sectional area, and muscle fibre types

Muscles were put onto a support made of cork and covered with tragacanth gum (Santa Cruz Biotechnology, Dallas, Tex, USA), before freezing in 2-methylbutane (M32631, Sigma-Aldrich, Saint-Louis, MO, USA) refrigerated with liquid nitrogen. Sections of 10 μm thickness were prepared using a cryostat (CM3050 S, Leica, Wetzlar, Germany). Muscle sections were incubated for 15 min at room temperature (RT), washed with PBS for 5 min, and permeabilised with 0.05% PBS-Triton-X100 three times for 5 min at room temperature (RT). Blocking was performed with 3% PBS-BSA for 1 h at RT. Each section was incubated with primary antibody for 1 h at RT in 3% PBS-BSA (Table S1). A coverslip (Diamant Star, Menzel-Gläser, Braunschweig, Germany) was mounted on each slide using fluoromonth (F4680, Sigma-Aldrich, St Louis, MO, USA). The sections were imaged by an automated slide scanner (Axioscan Z1, Zeiss, Oberkochen, Germany). Images were automatically analysed using the MuscleJ plugin with Image J software to obtain phenotyping and cross-sectional-area data.

### Treadmill

The mouse was placed on a static treadmill for 2 min for acclimation. The treadmill was set at 10 m/min for 10 min, and the mouse was left on the treadmill for 2 min before being replaced in its cage. The effort test was carried out 1 h after acclimation. The mouse was placed on a static treadmill for 2 min, then the treadmill was programmed to run at 10 m/min, at the same time a chronometer was started. Every 4 min, the speed was increased by 4 m/min until the mouse was exhausted. Exhaustion was considered to have occurred when the mouse remained on the electric grid for 5 consecutive seconds. The experimental procedure was performed under blinded conditions.

### Ex vivo contractility

Muscle contractility was performed by using the DMT Muscle Strip Myograph system, coupled to the CS4 + stimulator (820MO, DMT, Hinnerup, Denmark). *Soleus* were dissected under regular irrigation with Krebs solution (120 mM NaCl, 4.7 mM KCl, 1.25 mM CaCl₂, 1.2 mM MgSO₄, 25 mM NaHCO₃, 1.2 mM KH₂PO₄, 11 mM D-glucose and 2 mM Na-pyruvate). Proximal and distal ligatures were made during dissection using 4.0 non-absorbable sutures. After isolation, muscles were placed in a contractility chamber containing the same Krebs solution that was oxygenated continuously with a Carbogen mixture (95% O₂ / 5% CO₂), and adjusted to pH 7.4. A stabilisation phase was performed at a maintained passive voltage of 10 mN. The optimal stimulation voltage was determined on the basis of the maximal contractile response to twitch stimulations (1 Hz). A supra-optimal voltage of 38 V (+ 20%) was then used for all analyses. The optimal length (L₀) was progressively adjusted to obtain the maximum force in response to twitch and then tetanic stimulation (150 Hz, 500 ms). Next, an automated stimulation protocol using MyoPULSE software was applied to analyse muscle contraction parameters. Muscle fatigability was assessed by continuous stimulation at 40 Hz for 120 s. The experimental procedure was performed under blinded conditions.

### C2C12 murine myoblast cell line

Murine myoblasts were obtained from the American Type Culture Collection (ATCC, CRL-1772, Manassas, VA, USA). Cells were cultured in high-glucose (4.5 g/L) Dulbecco’s modified eagle medium (DMEM – Thermo Fisher Scientific, Waltham, MA, USA), supplemented with 10% fetal bovine serum (FBS – Thermo Fisher Scientific, Waltham, MA, USA) and 1% penicillin/streptomycin (P/S – Thermo Fisher Scientific). When confluence reached 90%, differentiation was induced by replacing the growth media with high-glucose DMEM, supplemented with 2% horse serum (HS – Dominique Dutsher, Bernolsheim, France) and 1% P/S. Cells were maintained in a humid atmosphere with 5% CO_2_ at 37 °C. Media changes were performed every 48–72 h. After 7 days of differentiation, cells were treated for 14 days with lipopolysaccharide (LPS, 100 ng/ml, L2880, Sigma-Aldrich, St Louis, MO, USA) or solvent (PBS).

### High-resolution respirometry on permeabilised muscle fibres

The PMG protocol enables assessment of the mitochondrial respiration driven by the different complexes sequentially, by complex I, complex II, and finally complex IV on *soleus* permeabilised fibres. *Soleus* were placed in BIOPS (2.77 mM, CaK_2_EGTA, 7.23 mM, K_2_EGTA, 0.5 mM, 6.56 mM MgCl_2_-6H_2_O, 15 mM Na_2_phosphocreatine, 0.5 mM dithiothreitol, 5.77 mM Na_2_ATP, 20 mM imidazole, 20 mM taurine, 50 mM MES-hydrate, pH 7.1) at 4 °C for mechanical dissociation. The resulting fibres were chemically permeabilised in 1.5 ml BIOPS containing saponin (50 µg/µL) at 4 °C for 30 min, with gentle agitation. Washed twice in Mir05 respiration buffer (60 mM lactobionic acid, 3 mM MgCl_2_-6H_2_O, 20 mM taurine, 20 mM HEPES, 110 mM sucrose, 10 mM KH_2_PO_4_, 0.5 mM EGTA, 1 g/L Bovine Serum Albumin (BSA) fatty acid-free, pH 7.1), muscle fibres (2–4 mg) were placed into the hyper oxygenated O2K chambers (400 nmol O_2_/L, Oroboros Instruments, Innsbruck, Austria) and oxygen consumption was measured at 25 °C. Two protocols were carried out. This first protocol was: (i) addition of pyruvate (5 mM), malate (2 mM), and glutamate (10 mM), to assess the oxygen consumption (JO_2_) when the electron transfer chain (ETC) is not coupled to the ATP synthase (noted LEAK); (ii) addition of ADP (5 mM) to evaluate the JO_2_ when the ETC fed with electrons from complex I (CI-CIV) is linked to the ADP synthase (oxidative phosphorylation condition-OXPHOS); (iii) injection of the complex I inhibitor Rotenone (0.5 µM) and succinate (10 mM) to assess the JO_2_ with electrons circulating from complex II to IV (CII-CIV) in OXPHOS conditions; (iv) addition of the complex III inhibitor antimycin A (2.5 µM) and addition of ascorbate (2 mM) and tetramethyl-p-phenylenediamine dihydrochloride (0.5 mM) to measure maximal JO_2_ of complex IV (CIV). The second protocol consisted of: (i) injection of octanoyl-L-carnitine (0.5 mM) and malate (2 mM) to assess LEAK; (ii) addition of ADP (5 mM) to measure JO_2_ in OXPHOS conditions. During each protocol, cytochrome c (10 µM) Q was added to test the outer mitochondrial membrane integrity: experiments were excluded if the increase in JO_2_ was higher than 15% following).

### High-resolution respirometry on muscle cells

Differentiated C2C12 cells (0.5 million/mL) suspended in differentiation media were placed into the O2K chambers. After stabilisation (basal respiration), the ATP synthase inhibitor oligomycin (10 µM) was added to assess the JO_2_ in the absence of ATP production (LEAK). Sequential Injections of the uncoupler 3-chlorophenylhydrazone carbonyl cyanide (CCCP – 1 µM per injection) were performed to obtain the maximal JO_2_ (maximal respiration). Finally, spare respiratory capacity was calculated as maximal respiration less basal respiration.

### Western-blot

*Quadriceps* was pulverised in powder using a cell crusher (Cellcrusher, Schull, Co. Cork, Ireland). RIPA lysis buffer (10 mM Tris-HCl pH 7.4, 5 mM EDTA, 0. 1% SDS, 150 mM NaCl, 1% sodium deoxycholate, 1% Triton), containing 1 mM phenylmethanesulfonyl fluoride (#36978, Thermo Fisher, MA, USA) and 1 mM protease/phosphatase inhibitor cocktail (#5872, Cell Signaling Technology, Danvers, MA, USA) was added to approximately 20 mg of muscle powder (60 µL/mg). Then, samples were homogenised using a Bead Mill (Thermo Fisher Scientific, Waltham, MA, USA) and centrifuged at 15,000 *g* for 10 min at 4 °C. The supernatant protein concentration was determined using Bradford’s reagent (B6916, Sigma, Burlington, VT, USA). 40 µg of protein were mixed into a solution with LDS and a reducing agent solution (B0008, B0009, Thermofisher, Waltham, MA, USA) and were denatured for 10 min at 70 °C. Proteins were then separated by electrophoresis in a Tris-glycine buffer (Tris 25 mM, glycine 192 mM, SDS 0.1%, pH 8.3) on 8–15% SDS polyacrylamide gels. Proteins were transferred to polyvinylidene difluoride (PVDF) membranes (Trans-Blot Turbo, Bio-Rad, Hercules, CA, USA) using the Trans-Blot Turbo Transfer System (Bio-Rad, Hercules, CA, USA). Membranes were incubated in 5% milk or BSA solution in Tris-Buffered Saline containing 0.1% Tween 20 (TBST) and then incubated overnight at 4 °C with the primary antibody (Table S1), followed by one-hour incubation with a secondary antibody conjugated to horseradish peroxidase or a fluorophore (Table S1). Protein complexes were revealed by chemiluminescence (Clarity Western ECL Substrate Kit, Bio-Rad, Hercules, CA, USA) or fluorescence (Odyssey Fx, Li-Cor, Lincoln, NE, USA). Protein expression was quantified using ImageJ software (NIH, Bethesda, MD, USA, https://imagej.nih.gov/ij/) and standardised with the glyceraldehyde 3-phosphate dehydrogenase (GADPH) protein.

### Gene expression with Quantigene

*Quadriceps* (~ 4 mg) were placed in 40 µL of working homogenisation solution (50 mg/mL proteinase K (QP1013) and homogenisation solution (QS0106) at a ratio of 1:100). Samples were incubated at 65 °C for 30 min, vortexed every 5 min until a homogeneous mixture was obtained, and used according to the manufacturer’s instructions (Thermo Fisher Scientific). Plates were read using a Bio-Plex (Bio-Rad, Hercules, CA, USA), the background noise was subtracted, and the values normalised to the geometric mean of the reference genes (Rpl23, Rpl32, and Pgk1).

### mtDNA copy number

Total DNA was isolated from 20 mg of quadriceps using the QIAamp Fast DNA tissue kit (QIAGEN, Hilden, Germany), according to the manufacturer’s protocol. 40 pg of total DNA was used to perform qPCR. qPCRs were performed and followed in real-time using the PowerUp SYBR Green Master Mix kit (Thermo Fisher, Waltham, MA, USA) and the QuantStudio 3 Real-Time PCR System (Applied Biosystems, Waltham, MA, USA), respectively. Relative quantification of ND1, a mitochondrial gene, and PPIA, a nuclear gene, was performed using method 2^−ΔΔCt^. The ratio of the mitochondrial NADH dehydrogenase 1 (ND1) gene to the nuclear peptidyl-prolyl cis-trans isomerase (PPIA) gene. Primers used are available in Table S2.

### Statistical analysis

Statistical analysis was performed using GraphPad Prism 10 (GraphPad, San Diego, CA, USA). Quantitative values are expressed as means ± standard error of the mean (SEM). Kaplan-Meier survival curves were compared using the log-rank test. After performing the Shapiro-Wilk normality test, comparisons between two groups were made with a Student’s t-test or Mann-Whitney test. Comparisons between three or more groups were performed by a one-way ANOVA test with a Sidak or Fisher’s post-hoc test if the values followed a normal distribution (Shapiro-Wilk test), otherwise a Kruskal-Wallis was performed with a Dunn’s post-hoc test. Comparisons of three or more groups using a mixed-effects model (REML).

## Supplementary Information

Below is the link to the electronic supplementary material.

Supplementary Material 1

## Data Availability

The datasets used and/or analysed during the current study are available from the corresponding author on reasonable request.
