# Supplementary material for: RAGE contributes to persistent sepsis-induced muscle and mitochondrial alterations
Source: Sci Rep. 2025 Nov 28;15:45768. doi: 10.1038/s41598-025-28645-8 (PMC12756265; doi:10.1038/s41598-025-28645-8)
Supplement: Supplementary file 1 — Supplementary Material 1 [file 41598_2025_28645_MOESM1_ESM.pdf]

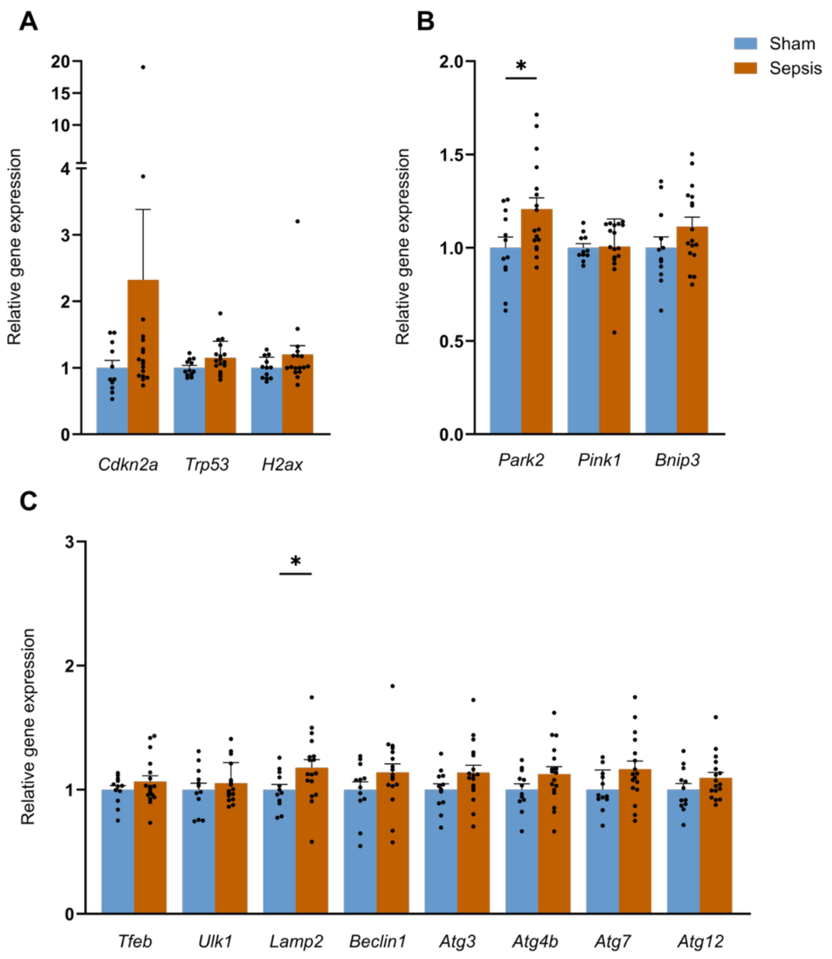

Figure S1. Analysis of senescence-, mitophagy- and autophagy-related genes. A, mRNA levels of *Cdkn2a, Trp53,* and *H2ax* by Quantigene in *quadriceps* for Sham (n=12) and Sepsis (n=17). **B**, mRNA levels of *Park2, Pink1,* and *Bnip3* by Quantigene in *quadriceps* for Sham (n=12) and Sepsis (n=17). **C**, mRNA levels of *Tfeb, Ulk1, Lamp2, Beclin1, Atg3, Atg4b, Atg7* and *Atg12* by Quantigene in *quadriceps* for Sham (n=12) and Sepsis (n=17). Data are expressed as mean with SEM. Statistical comparisons between Sham vs Sepsis (*). Data were analysed with a Mann-Whitney test (**A**, **B**) or with a t-test (**B**,**C**), according to the results of the normality test. * $p < 0.05$.

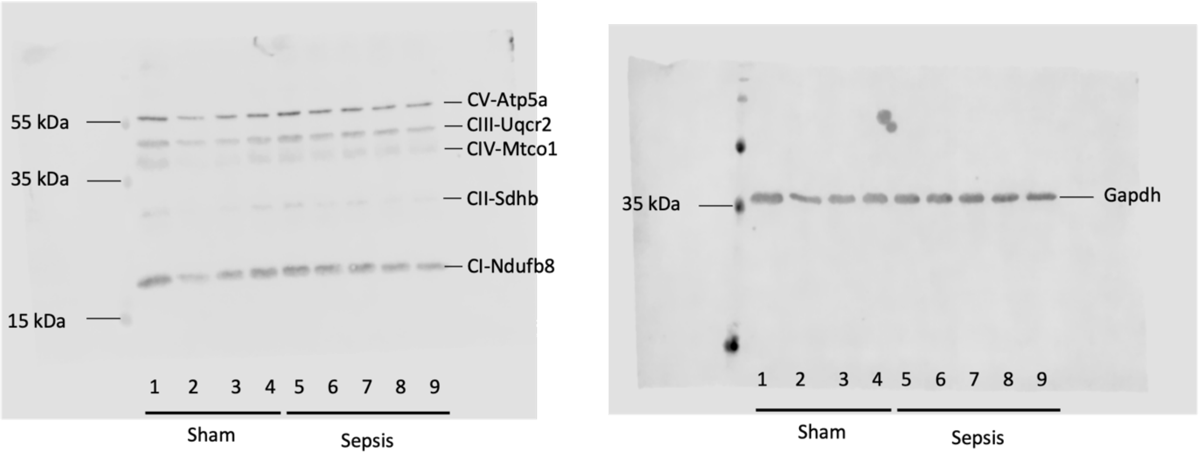

Figure S2. Full-length western blot of OXPHOS (Ndufb8, Sdhb, Uqcr2, Mtco1, Atp5a-left) and Gadph (right) with molecular weight markers indicated, corresponding to Figure **3F** in the main manuscript. Lanes correspond to Sham and Sepsis groups as shown. Exposure time: 2 min.

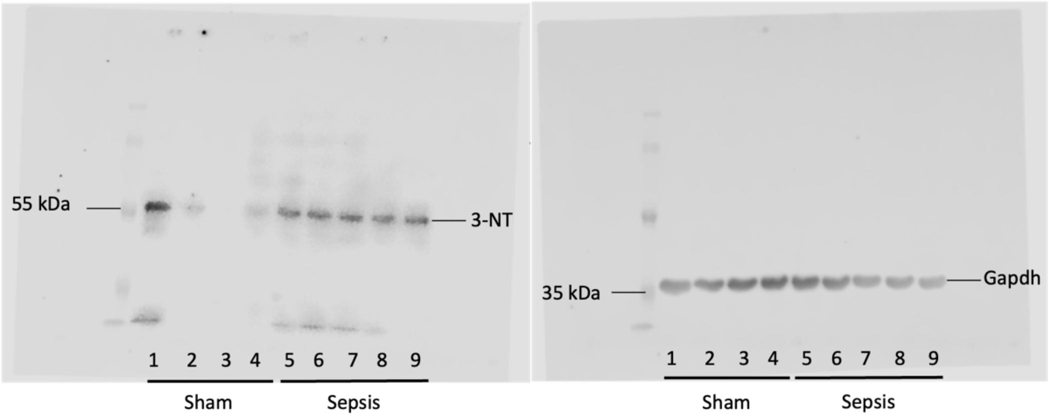

Figure S3. Full-length western blot of 3-Nitrotyrosine (left) and Gadph (right) with molecular weight markers indicated, corresponding to Figure **3I** in the main manuscript. Lanes correspond to Sham and Sepsis groups as shown. Exposure time: 2 min.

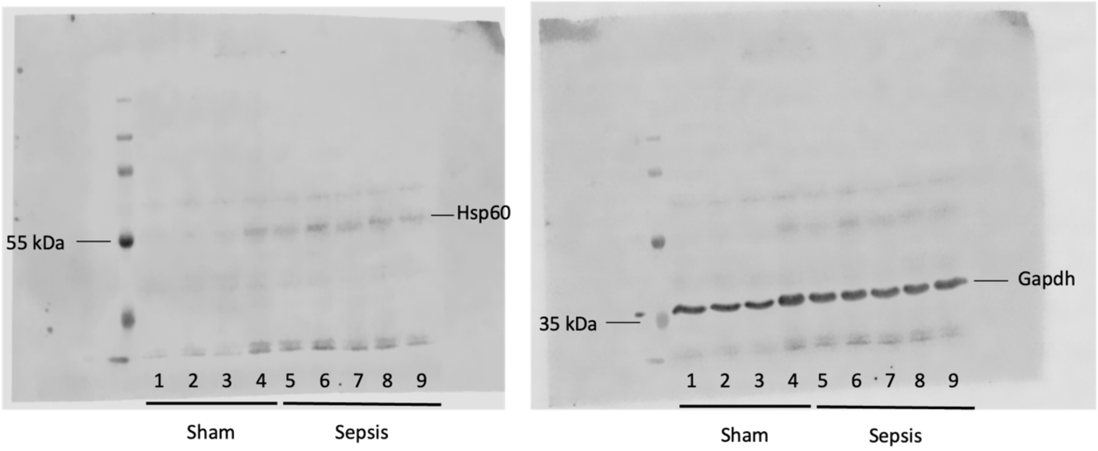

Figure S4. Full-length western blot of Hsp60 (left) and Gadph (right) with molecular weight markers indicated, corresponding to Figure **3J** in the main manuscript. Lanes correspond to Sham and Sepsis groups as shown. Exposure time: 2 min.

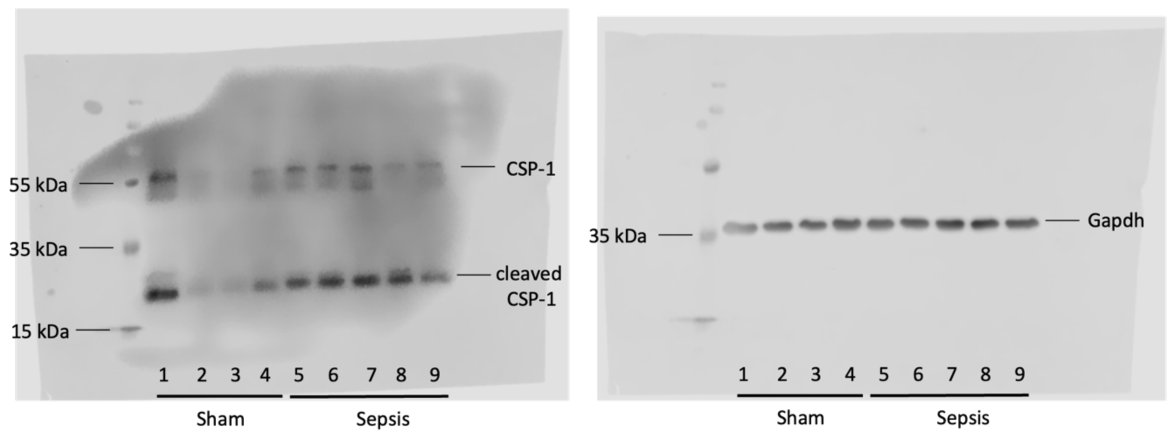

Figure S5. Full-length western blot of cleaved CSP-1 (left) and Gadph (right) with molecular weight markers indicated, corresponding to Figure **4B** in the main manuscript. Lanes correspond to Sham and Sepsis groups as shown. Exposure time: 2 min.

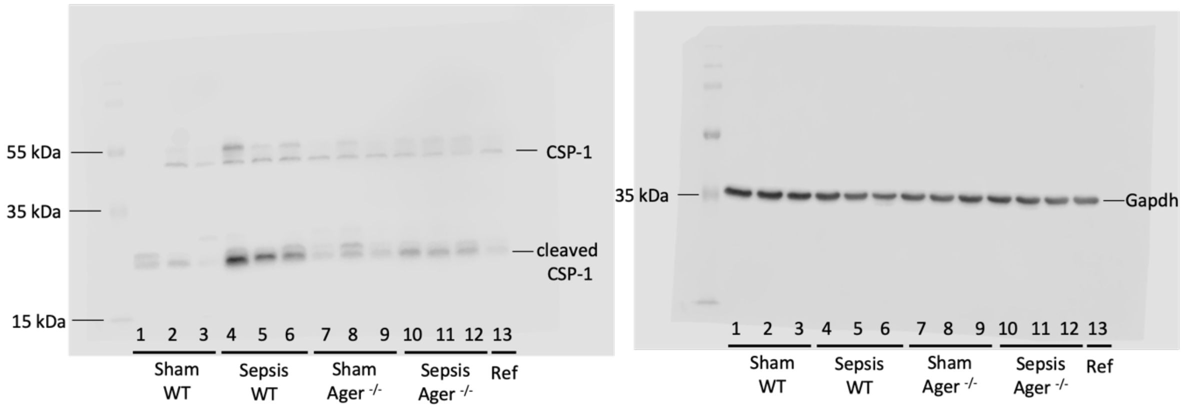

Figure S6. Full-length western blot of cleaved-CSP1 (left) and Gadph (right) with molecular weight markers indicated, corresponding to Figure **6B** in the main manuscript. Lanes correspond to Sham WT, Sepsis WT, Sham Ager $^{-/-}$ and Sepsis Ager $^{-/-}$ groups as shown. Exposure time: 2 min.

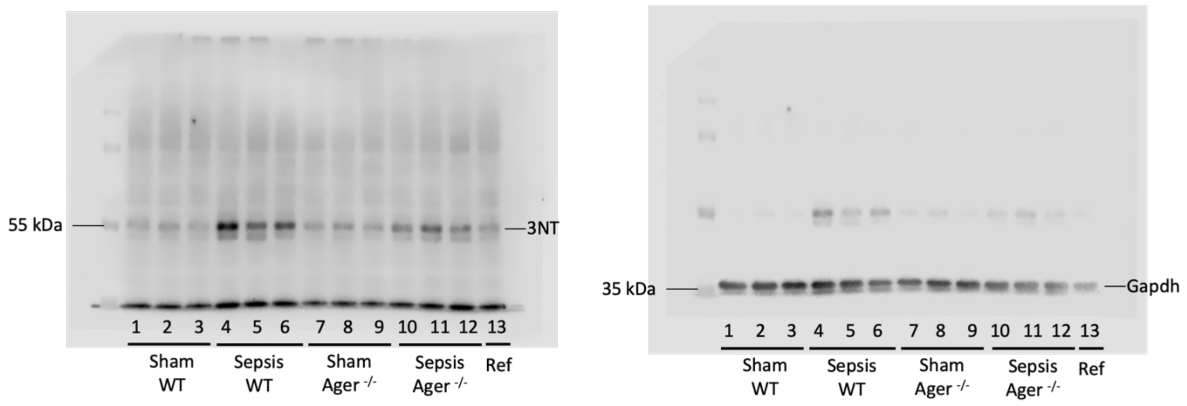

Figure S7. Full-length western blot of 3-NT (left) and Gadph (right) with molecular weight markers indicated, corresponding to Figure **6C** in the main manuscript. Lanes correspond to Sham WT, Sepsis WT, Sham Ager $^{-/-}$ and Sepsis Ager $^{-/-}$ groups as shown. Exposure time: 2 min.

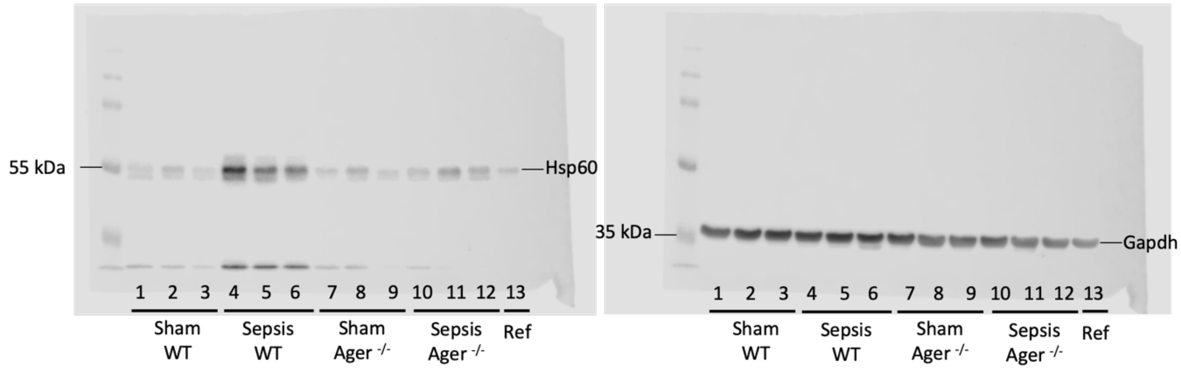

Figure S8. Full-length western blot of Hsp60 (left) and Gadph (right) with molecular weight markers indicated, corresponding to Figure **6D** in the main manuscript. Lanes correspond to Sham WT, Sepsis WT, Sham Ager $^{-/-}$ and Sepsis Ager $^{-/-}$ groups as shown. Exposure time: 2 min.

Target	Reference	Supplier	Dilution
**Primary antibody**			
OXPHOS	stn-19467	Abcam	1/3000
3-NT	ab-61392	Abcam	1/3000
Hsp60	sc-59567	Santa Cruz Biotechnology	1/3000
Csp-1	AG-20B-0042-C100	AdipoGen Life Sciences	1/3000
MHC IIa	SC-71	DSHB	1/100
MHC IIb	BF-F3	DSHB	1/50
MHC I	BA-D5	DSHB	1/50
Laminin	4h8-2	DSHB	1/100
**Secondary Antibody**			
Mouse IgG 700	926-68170	Li-Cor	1/10000
Mouse IgG 800	925-32212	Li-Cor	1/10000
Rabbit IgG 700	926-68071	Li-Cor	1/10000
Rabbit IgG 800	827-08365	Li-Cor	1/10000
Mouse IgG1 (Alexa Fluor 488)	A-21121	Thermofisher	1/250
Mouse IgM (Alexa Fluor 555)	A-21426	Thermofisher	1/250
Mouse IgG2b (Alexa Fluor 647)	A-21242	Thermofisher	1/250
Rat IgG (Alexa Fluor 555)	A-48261	Thermofisher	1/250
Mouse IgG-HRP-linked	7076	Cell signaling	1/5000
Rabbit IgG-HRP-linked	7074	Cell signaling	1/5000

Table S1. Antibodies used in western blot and immunofluorescence experiments.

	Forward sequence	Reverse sequence	Accession number
Nd1	TCCGAGCATCTTATCCACGC	GTATGGTGGTACTCCCGCTG	NC_005089.1
Ppia	ACACGCCATAATGGCACTGG	CAGTCTTGGCAGTGCAGAT	NM_008907.2

Table S2. Primer sequences used for qPCR.
